# Melatonin inhibits triple-negative breast cancer progression through the Lnc049808-FUNDC1 pathway

**DOI:** 10.1038/s41419-021-04006-x

**Published:** 2021-07-16

**Authors:** Anli Yang, Fu Peng, Lewei Zhu, Xing Li, Shunling Ou, Zhongying Huang, Song Wu, Cheng Peng, Peng Liu, Yanan Kong

**Affiliations:** 1 Sun Yat-sen University Cancer Center, State Key Laboratory of Oncology in South China, Collaborative Innovation Center for Cancer Medicine, Guangzhou, P. R. China; 2 Key Laboratory of Systematic Research of Distinctive Chinese Medicine Resources in Southwest China, Chengdu University of Traditional Chinese Medicine, Chengdu, P. R. China; 3 West China School of Pharmacy, Sichuan University, Chengdu, P. R. China; 4 Department of Breast Surgery, The First People’s Hospital, Foshan, Guangdong People’s Republic of China

**Keywords:** Breast cancer, Non-coding RNAs

## Abstract

Melatonin has been reported to have tumor-suppressive effects via comprehensive molecular mechanisms, and long non-coding RNAs (lncRNAs) may participate in this process. However, the mechanism by which melatonin affects the function of lncRNAs in triple-negative breast cancer (TNBC), the most aggressive subtype of breast cancer, is still unknown. Therefore, we aimed to investigate the differentially expressed mRNAs and lncRNAs in melatonin-treated TNBC cells and the interaction mechanisms. Microarray analyses were performed to identify differentially expressed mRNAs and lncRNAs in TNBC cell lines after melatonin treatment. To explore the functions and underlying mechanisms of the mRNAs and lncRNAs candidates, a series of in vitro experiments were conducted, including CCK-8, Transwell, colony formation, luciferase reporter gene, and RNA immunoprecipitation (RIP) assays, and mouse xenograft models were established. We found that after melatonin treatment, FUNDC1 and lnc049808 downregulated in TNBC cell lines. Knockdown of FUNDC1 and lnc049808 inhibited TNBC cell proliferation, invasion, and metastasis. Moreover, lnc049808 and FUNDC1 acted as competing endogenous RNAs (ceRNAs) for binding to miR-101. These findings indicated that melatonin inhibited TNBC progression through the lnc049808-FUNDC1 pathway and melatonin could be used as a potential therapeutic agent for TNBC.

## Introduction

Triple-negative breast cancer (TNBC) accounts for 15–20% of all breast cancers, and compared with other subtypes, it exhibits more aggressive biological behaviors and worse clinical outcomes due to the lack of efficient molecular targets [1]. Melatonin (N-acetyl-5-methoxytryptamine) is a natural indoleamine, which is mainly produced by the pineal gland in humans and other animals in response to darkness and light [2, 3]. Studies have shown that in addition to circadian rhythm monitoring, melatonin also has anti-inflammatory, antioxidant, immunomodulatory, vascular regulation, and anticancer activities [4–7]. The anticancer properties of melatonin are mediated via suppression of tumor metabolism and critical signaling pathways, including PI3K/Akt, NF-kB, hypoxia-inducible factor-1 (HIF-1), cyclin-dependent kinase (CDKs), insulin-like growth factor receptor (IGF-1R), and estrogen receptor signaling [8–10]. A series of studies indicated that melatonin suppressed ERα mRNA expression, inhibited p38 MAPK signaling, repressed epithelial-to-mesenchymal transition, and correlated with intrinsic resistance to tamoxifen and doxorubicin in estrogen receptor alpha (ERα)-positive human breast cancer [11, 12]. However, the role of melatonin in TNBC remains controversial. Specifically, it is unclear how long noncoding RNAs (lncRNAs) are involved in regulating TNBC.

LncRNAs, more than 200 nucleotides, are a type of transcript without protein translation. It has been proven that they play oncogenic or tumor-suppressive roles in various malignancies and are involved in the progression of breast cancer [13–15]. However, whether melatonin affects lncRNA expression and the mechanisms underlying the effects of melatonin in TNBC remain unknown. Therefore, our study aims to explore the expression profiles of mRNAs and lncRNAs in melatonin-treated TNBC cells by whole-genome mRNA and lncRNA expression microarray analyses. Moreover, a series of in vitro and in vivo experiments were conducted to further explore the roles and mechanisms of the screened differentially expressed mRNAs and lncRNAs in TNBC. Our results revealed that FUNDC1, a highly conserved mitochondrial outer membrane protein that plays an important role in mitochondrial autophagy, and the lnc049808 (NONMMUT049808) significantly downregulated in TNBC cell lines that were pretreated with melatonin. Knockdown of FUNDC1 and lnc049808 suppressed TNBC cell proliferation, invasion, and metastasis. Functional assays showed that lnc049808 and FUNDC1 acted as competing endogenous RNAs (ceRNAs) for binding to miR-101. Our findings indicated that melatonin inhibited TNBC progression through the lnc049808-FUNDC1 pathway and that melatonin could be used as a potential therapeutic agent for TNBC.

## Results

### FUNDC1 knockdown inhibited TNBC progression

**Fig. 1:**
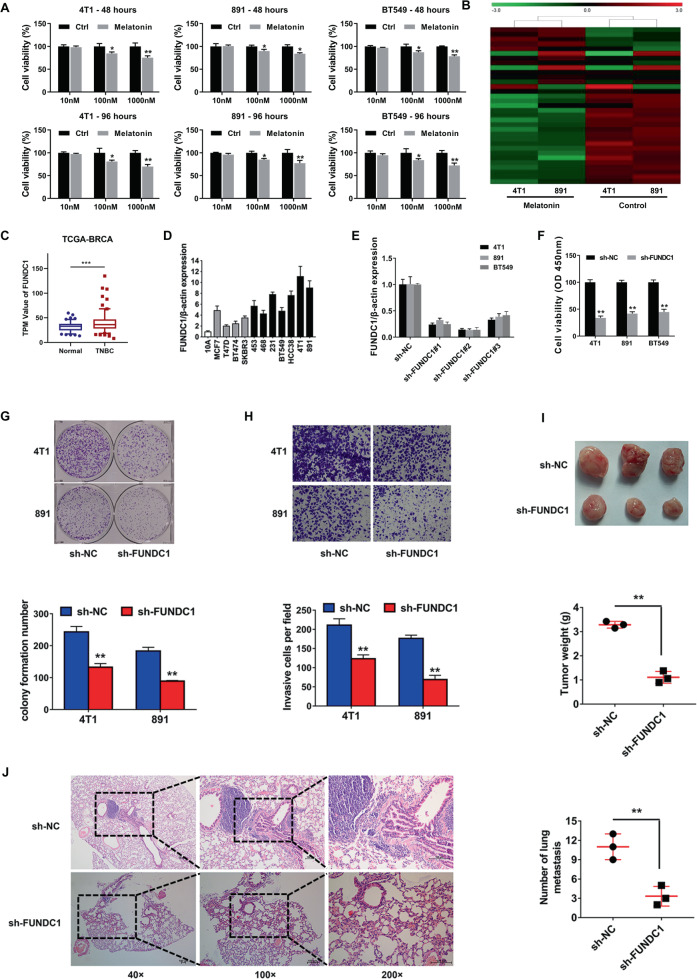
FUNDC1 knockdown inhibited TNBC Progression. **A** 4T1, 891, and BT549 TNBC cells were treated with melatonin (10–1000 nM) or the vehicle for 48 or 96 h. **B** Whole-genome mRNA expression microarray analyses were performed on melatonin-treated and melatonin-untreated 4T1 and 891 cells. **C** FUNDC1 was upregulated in TNBC tissues compared to normal tissues, according to the TCGA database. **D** The expression levels of FUNDC1 were determined in breast cancer cell lines by qRT-PCR. White: normal breast epithelial cell line; gray: non-TNBC cell line; black, TNBC cell line. **E** qRT-PCR showed that FUNDC1 knockdown was successful with sh-FUNDC1#2. **F** A CCK-8 assay was performed after transfection in 4T1, 891, and BT549 TNBC cells. **G** A colony formation assay was performed (upper), and the results were quantified (lower). **H** A Transwell assay was performed (upper), and the results were quantified (lower). **I** Representative images of xenograft tumors are shown (upper), and tumor weight was quantified (lower, *n* = 3 per group). **J** H&E-stained sections of metastatic lung nodules are shown (left), and the number of metastatic nodules was quantified (right, *n* = 3 per group). *p < 0.05, **p < 0.01.

To validate the effect of melatonin on TNBC, 4T1, 891, and BT549 TNBC cells were treated with melatonin (10–1000 nM) or the vehicle for 48 or 96 h (Fig. 1A). We found that melatonin inhibits TNBC cell proliferation in a dose- and time-dependent manner. To reveal the underlying mechanism, whole-genome mRNA expression microarray analyses were performed in melatonin-untreated and melatonin-treated 4T1 and 891 cells. The top 33 differentially expressed mRNAs are shown in Fig. 1B. Among these mRNAs, FUNDC1 was upregulated in the melatonin-treated cells, which was a highly conserved mitochondrial outer membrane protein and played an important role in mitochondrial autophagy. However, the expression and function of FUNDC1 are unknown in TNBC. According to the TCGA database, FUNDC1 was increased in TNBC tissues compared to normal tissues (Fig. 1C). We further confirmed that the expression levels of FUNDC1 upregulated in breast cancer cell lines (Fig. 1D). To explore the function of FUNDC1 in the progression of TNBC, we used short hairpin RNA (shRNA) to knock down FUNDC1 expression. Three candidate shRNAs were constructed, and their knockdown efficiency was validated. We found that sh-FUNDC1#2 produced the strongest inhibition, and this shRNA was selected for use in the following experiments (Fig. 1E). The Cell Counting Kit-8 (CCK-8) assay revealed significant inhibition of cell proliferation by FUNDC1 knockdown (Fig. 1F). FUNDC1 knockdown also weakened the cell colony formation ability (Fig. 1G). Transwell assay showed that FUNDC1 knockdown suppressed cell invasion (Fig. 1H). To further confirm the function of FUNDC1 in vivo, we established mouse xenograft models and found that FUNDC1 knockdown significantly suppressed tumor growth (Fig. 1I) and lung metastasis (Fig. 1J). Our findings demonstrated that FUNDC1 knockdown inhibited TNBC progression.

### FUNDC1 is a target gene regulated by miR-101

**Fig. 2:**
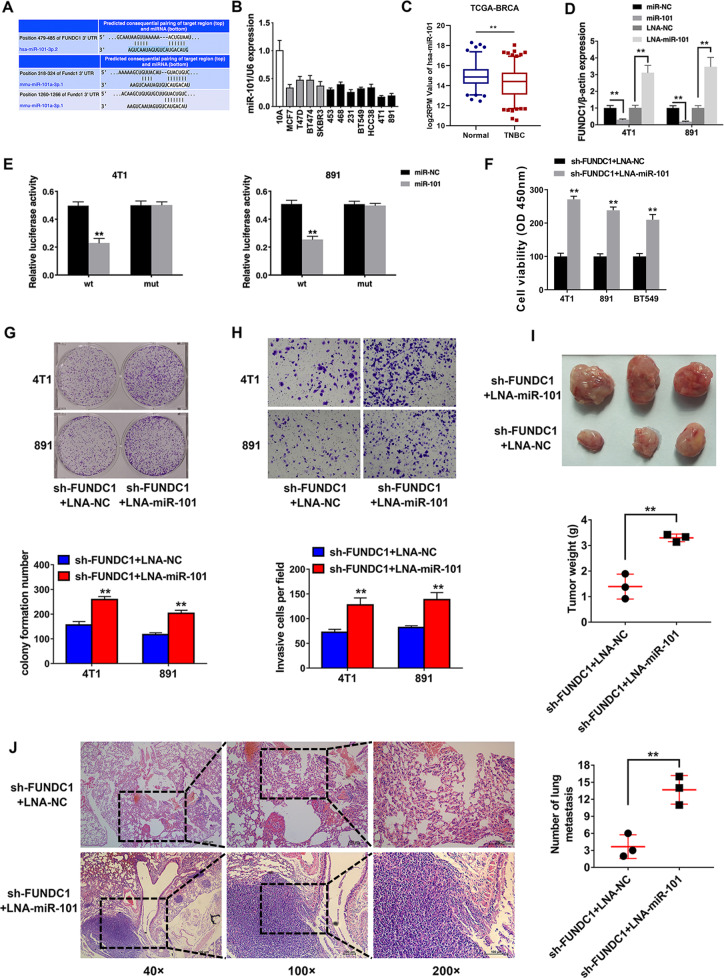
FUNDC1 Is a Target Gene regulated by miR-101. **A** TargetScan showed the predicted binding sites of miR-101 within FUNDC1. **B** The expression of miR-101 was detected in breast cancer cell lines. White: normal breast epithelial cell line; gray: non-TNBC cell line; black, TNBC cell line. **C** hsa-miR-101 was downregulated in TNBC tissues compared to normal tissues, according to the TCGA database. **D** The expression level of FUNDC1 after transfection with the miR-101 mimic or LNA-miR-101 was determined by qRT-PCR. **E** Luciferase assay of cells transfected with vectors containing the FUNDC1 3′ UTR (WT) or its mutant (mut). **F** A CCK-8 assay was performed after transfection in 4T1, 891, and BT549 TNBC cells. **G** A colony formation assay was performed (upper), and the results were quantified (lower). **H** A Transwell assay was performed (upper), and the results were quantified (lower). **I** Representative images of xenograft tumors were shown (upper), and tumor weight was quantified (lower, *n* = 3 per group). **J** H&E-stained sections of metastatic lung nodules were shown (left), and the number of metastatic nodules was quantified (right, *n* = 3 per group). *p < 0.05, **p < 0.01.

To figure out the potential microRNAs (miRNAs) that may regulate FUNDC1, we searched the TargetScan, and miR-101 was predicted (Fig. 2A). MiR-101 serves as a tumor suppressor and participate in multiple cancer-related biological processes, including proliferation, apoptosis, angiogenesis, drug resistance, invasion, and metastasis. To validate our bioinformatics prediction, we measured the expression levels of miR-101 and found it was downregulated in TNBC cell lines (Fig. 2B). Consistently, the hsa-miR-101 was also downregulated in TNBC tissues compared to normal tissues, according to the TCGA database (Fig. 2C). Besides, we observed that FUNDC1 was downregulated by miR-101 but upregulated by locked nucleic acid (LNA)-miR-101 (Fig. 2D). A luciferase reporter assay was performed to confirm the direct binding between FUNDC1 and miR-101. We found that the luciferase activity significantly decreased with the transfection of wild-type (WT) luciferase vector. However, no similar effect was observed with the transfection of mutant vector (Fig. 2E). These results suggested that FUNDC1 is a target gene regulated by miR-101. The CCK-8 assay revealed that miR-101 suppression reversed the inhibition of cell proliferation induced by FUNDC1 knockdown (Fig. 2F). MiR-101 suppression also reversed the decrease in the cell colony formation ability induced by FUNDC1 knockdown (Fig. 2G). The Transwell assay showed that miR-101 suppression reversed the increase in apoptosis and the decrease in cell invasion induced by FUNDC1 knockdown (Fig. 2H). The mouse xenograft experiments showed that the suppression of tumor growth (Fig. 2I) and lung metastasis (Fig. 2J) induced by FUNDC1 knockdown was also reversed by miR-101 suppression. These findings prove that FUNDC1 is a target gene regulated by miR-101.

### Lnc049808 knockdown inhibited TNBC progression

**Fig. 3:**
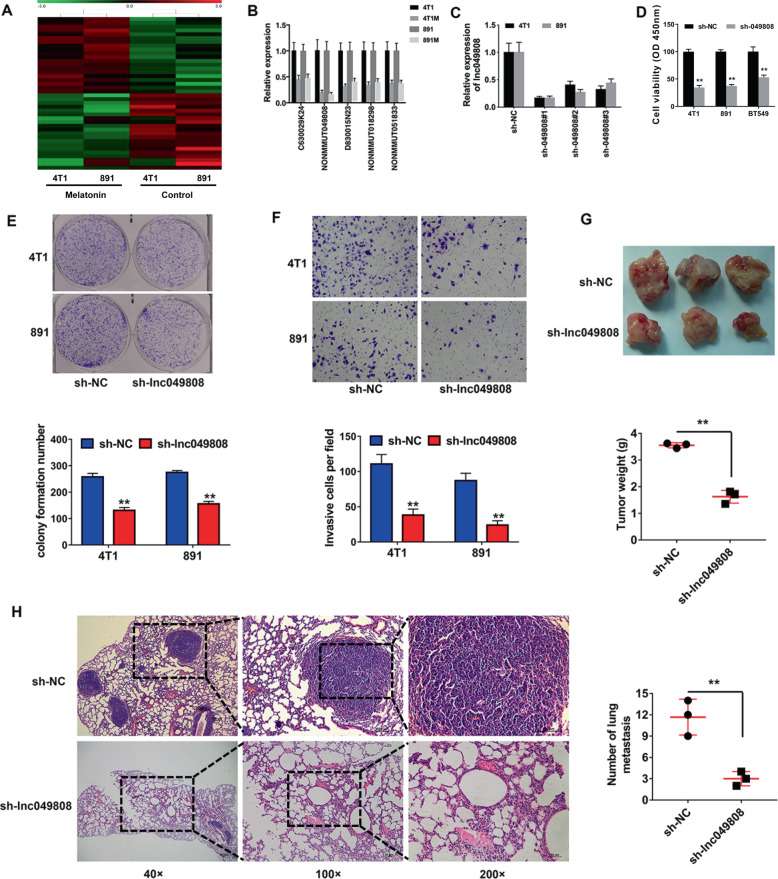
Lnc049808 knockdown inhibited TNBC progression. **A** LncRNA microarray analyses were performed on 4T1 and 891 melatonin-treated breast cancer cells. **B** The expression levels of the top five overlapping downregulated lncRNAs were determined by qRT-PCR. **C** qRT-PCR showed that lnc049808 knockdown was successful with sh-049808#1. **D** A CCK-8 assay was performed after transfection in 4T1, 891, and BT549 TNBC cells. **E** A colony formation assay was performed (upper), and the results were quantified (lower). **F** A Transwell assay was performed (upper), and the results were quantified (lower). **G** Representative images of xenograft tumors were shown (upper), and tumor weight was quantified (lower, *n* = 3 per group). **H** H&E-stained sections of metastatic lung nodules were shown (left), and the number of metastatic nodules was quantified (right, *n* = 3 per group). *p < 0.05, **p < 0.01.

LncRNA microarray analyses were performed with 4T1 and 891 TNBC cells pretreated with melatonin. The results showed that 40 lncRNAs downregulated at least twofold (Fig. 3A). Among these 40 lncRNAs, we validated the expression levels of the top five downregulated lncRNAs and found that NONMMUT049808 was the sharpest drop after melatonin treatment (Fig. 3B). Thus, we named this lncRNA lnc049808 and aimed to study it through a series of experiments. shRNA was used to knock down lnc049808 to explore the function of lnc049808 in TNBC progression, and sh-049808#1 produced the highest suppression efficiency and was adopted in subsequent experiments (Fig. 3C). The CCK-8 assay revealed significant inhibition of cell proliferation by lnc049808 knockdown (Fig. 3D). lnc049808 knockdown also weakened the cell colony formation ability (Fig. 3E). The Transwell assay showed that lnc049808 knockdown suppressed cell invasion (Fig. 3F). To further confirm the role of lnc049808 in vivo, mouse xenograft models were established, and significant inhibition of tumor growth (Fig. 3G) and lung metastasis by lnc049808 knockdown was observed (Fig. 3H). Our results showed that lnc049808 knockdown inhibited TNBC progression.

### Lnc049808 is a target of miR-101

**Fig. 4:**
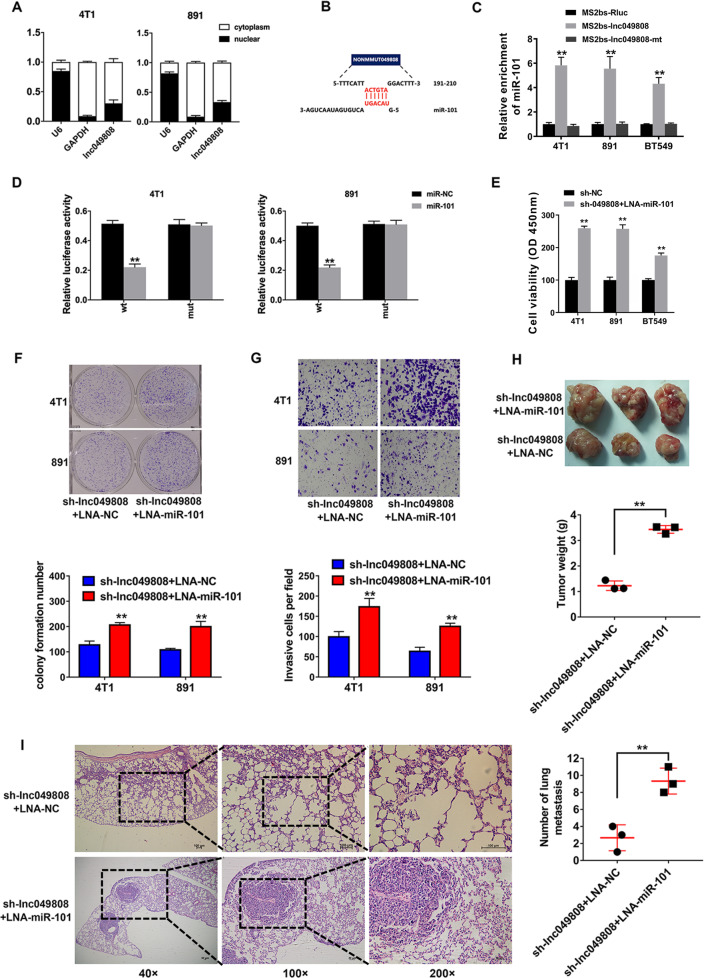
Lnc049808 Is a target of miR-101. **A** U6, GAPDH, and lnc049808 levels were determined by qRT-PCR. **B** The predicted binding sites of miR-101 within lnc049808 are shown. **C** The MS2-based RIP assay confirmed the binding of miR-101 and lnc049808 in 4T1, 891, and BT549 TNBC cells. **D** Luciferase assay of cells transfected with vectors containing binding sites for miR-101 within lnc049808 (WT) or its mutant (mut). **E** CCK-8 assay was performed after transfection in 4T1, 891, and BT549 TNBC cells. **F** A colony formation assay was performed (upper), and the results were quantified (lower). **G** A Transwell assay was performed (upper), and the results were quantified (lower). **H** Representative images of xenograft tumors were shown (upper), and tumor weight was quantified (lower, *n* = 3 per group). **I** H&E-stained sections of metastatic lung nodules were shown (left), and the number of metastatic nodules was quantified (right, *n* = 3 per group). *p < 0.05, **p < 0.01.

To explore the intracellular localization of lnc049808, we detected the expression of lnc049808 and found that lnc049808 was mainly localized in the cytoplasm (Fig. 4A). Via bioinformatic analysis, we found that lnc049808 contains sequences complementary to miR-101 (Fig. 4B). Next, we performed an RNA immunoprecipitation (RIP) assay and found that miR-101 could bind to lnc049808 (Fig. 4C). Furthermore, by conducting a luciferase reporter assay, decreased luciferase activity was observed after transfection with miR-101 (Fig. 4D), suggesting that lnc049808 is a target of miR-101. The CCK-8 assay revealed that miR-101 suppression reversed the inhibition of cell proliferation induced by knockdown of lnc049808 (Fig. 4E). MiR-101 suppression also reversed the decrease in the cell colony formation ability induced by lnc049808 knockdown (Fig. 4F). The Transwell assay showed that miR-101 suppression reversed the increase in apoptosis and the decrease in cell invasion induced by lnc049808 knockdown (Fig. 4G). The mouse xenograft experiments showed that the reduction in tumor growth (Fig. 4H) and lung metastasis (Fig. 4I) induced by lnc049808 knockdown could be reversed by miR-101 suppression. These findings proved that lnc049808 is a target of miR-101 and that miR-101 regulates the function of lnc049808.

### Lnc049808 and FUNDC1 function as ceRNAs to regulate miR-101

**Fig. 5:**
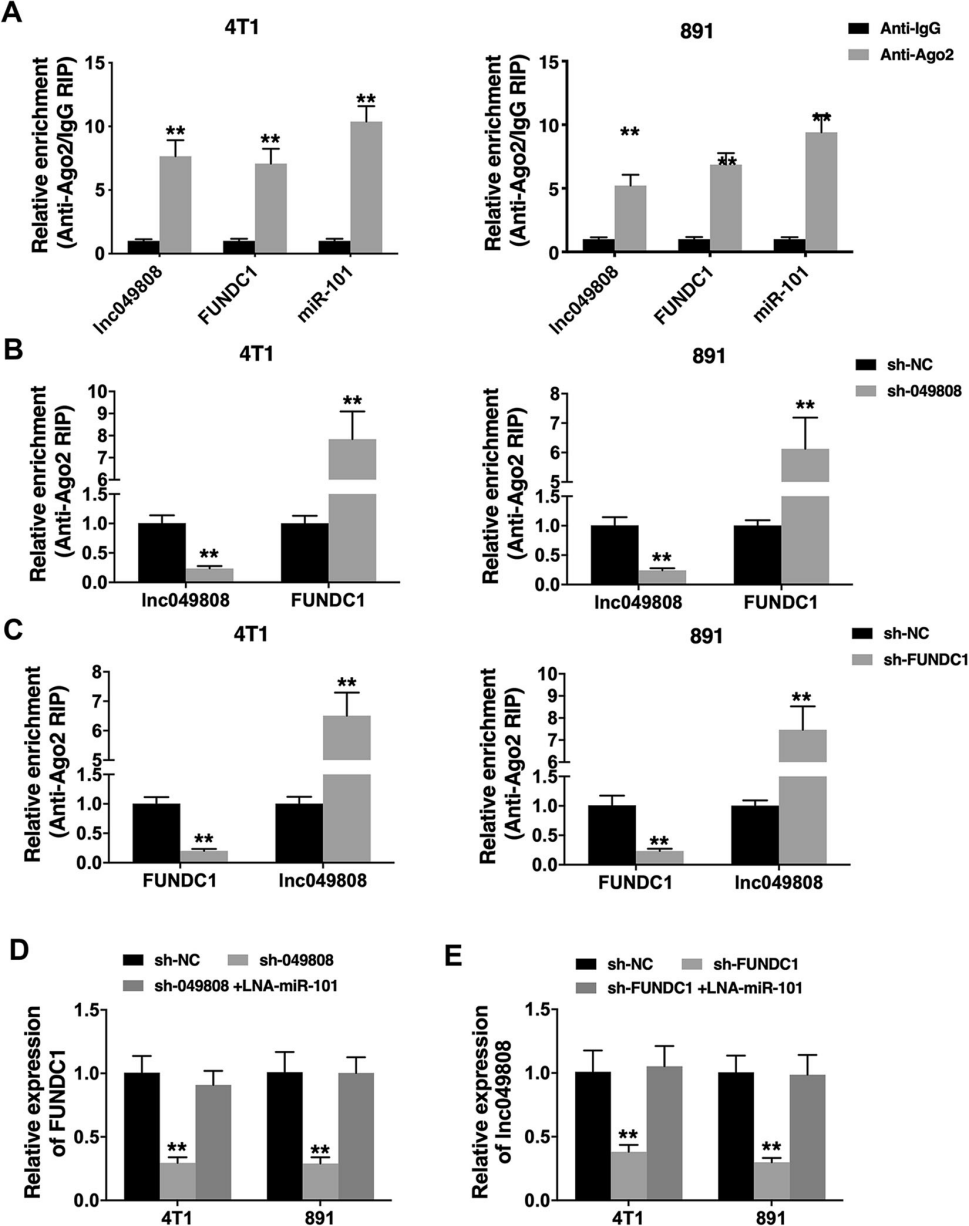
Lnc049808 and FUNDC1 function as ceRNAs to regulate miR-101. **A** The RIP assay showed the enrichment of Lnc049808, FUNDC1, and miR-101 on Ago2 relative to IgG in 4T1 and 891 TNBC cells. **B** Ago2-related RIP assay was conducted after transfection with sh-049808 vector. **C** Ago2-related RIP assay was conducted after transfection with sh-FUNDC1 vector. Enrichment of FUNDC1 to Ago2 RNA binding protein was decreased after FUNDC1 suppression. **D** The FUNDC1 level was quantified after transfected with sh-049808 vector and/or LNA-miR-101. **E** Cells were transfected with sh-049808 vector and/or LNA-miR-101, and the lnc049808 level was determined by qRT-PCR. **p < 0.01.

To explore the relationship among lnc049808, FUNDC1, and miR-101, we performed an Ago2 RIP assay and found that lnc049808, FUNDC1, and miR-101 were mainly enriched in the Ago2 precipitate (Fig. 5A). Furthermore, lnc049808 knockdown reduced the enrichment of lnc049808 on Ago2 but enhanced the enrichment of FUNDC1 on Ago2 (Fig. 5B). FUNDC1 knockdown reduced the enrichment of FUNDC1 on Ago2 but enhanced the enrichment of lnc049808 on Ago2 (Fig. 5C). These results suggested that lnc049808 and FUNDC1 can act as ceRNAs and compete for binding to miRNAs. The regulation of lnc049808, FUNDC1, and miR-101 was further confirmed, and we found that lnc049808 knockdown decreased FUNDC1 expression but LNA-miR-101 reversed the expression of FUNDC1 (Fig. 5D). We also observed decreased expression of lnc049808 when FUNDC1 was knocked down but increased expression of lnc049808 when miR-101 was inhibited (Fig. 5E). These data suggested that lnc049808 and FUNDC1 function as ceRNAs for miR-101 in regulating TNBC progression.

## Discussion

Melatonin has been reported to have anticancer effects via comprehensive molecular mechanisms, and lncRNAs may participate in this process. In this study, whole-genome mRNA and lncRNA expression microarray analyses were performed in melatonin-treated TNBC cells, and the top 20 significantly differentially expressed genes and lncRNAs were identified. Among the downregulated mRNAs in melatonin-treated TNBC cells, we identified FUNDC1, which is a highly conserved mitochondrial outer membrane protein and plays an important role in mitochondrial autophagy [16]. Many factors can induce mitophagy, including hypoxia, DNA damage, inflammation, nutrient deprivation, and mitochondrial membrane depolarization. Dysfunction in mitophagy affects cell metabolism and differentiation, leading to multiple types of diseases, including Alzheimer’s disease, heart failure, Parkinson’s disease, and cancers [17–19]. Although a previous study proved that FUNDC1 regulates mitochondrial dynamics and mitophagy in mammalian cells, the expression status and role of FUNDC1 in breast cancer are unclear. Here, we found that FUNDC1 upregulated in breast cancer cell lines and that knockdown of FUNDC1 suppressed breast cancer cell proliferation. Further exploration revealed that FUNDC1 was a target gene of miR-101 and that miR-101 regulated the expression and function of FUNDC1.

Recently, miR-101 has been proven to be a tumor suppressor in the initiation and progression of various malignancies [20]. Downregulation of miR-101 has been found in several malignancies, such as gastric cancer (GC) [21], hepatocellular carcinoma (HCC) [22], non-small-cell lung cancer (NSCLC) [23], cervical cancer [24], intrahepatic cholangiocarcinoma (ICC) [25], osteosarcoma (OS) [26], oral squamous cell carcinoma (OSCC) [27], bladder transitional cell carcinoma (BTCC) [28], and intraductal papillary mucinous neoplasm of the pancreas (IPMN) [29]. In breast cancer, miR-101 has been reported to act as a suppressor of cell proliferation by decreasing the level of DNA methyltransferase 3A (DNMT3A) and targeting proteasome maturation protein (POMP) and Stathmin 1 (Stmn1) [30, 31]. Our study showed that miR-101 inhibited TNBC progression by mediating FUNDC1, which confirmed the suppressive role of miR-101 in breast cancer.

Competing endogenous RNA (ceRNA) constitutes a microRNA-mediated mechanism by which RNAs mediate each other by binding to shared miRNAs [32]. A variety of forms of RNAs, including pseudogenes, protein-coding transcripts, circular RNAs, and lncRNAs, can act as ceRNAs. Many lncRNAs have been demonstrated to function as ceRNAs [33–36]. Lnc-ceRNAs are important regulators of tissue development processes, including muscle differentiation [33], embryonic stem cell self-renewal, and cancer progression [35]. Though lncRNAs are expressed at low levels, their expression is more tissue-specific than that of protein-coding genes [37]. As the first proven lnc-ceRNA, the HULC was one of the most significantly upregulated transcripts in HCC [38]. In the current study, we performed lncRNA microarray analyses and found that lnc049808 was downregulated in melatonin-treated breast cancer cells. Further investigation revealed that lnc049808 and FUNDC1 functioned as ceRNAs and competed for binding with miR-101 in the regulation of TNBC progression.

The antiproliferative effects of melatonin have been well studied in estrogen receptor α (ERα)-positive human breast cancer cell lines [39]. Previous studies revealed that melatonin suppresses ERα mRNA expression and ERα transcriptional activity via the MT1 receptor. However, the role of melatonin in ERα-negative cell lines is controversial. Mao et al. reported that the MDA-MB-231 human breast cancer cell line was unresponsive to melatonin’s antiproliferative effect in vitro [40]. Kim et al. demonstrated that melatonin did not affect proliferation but suppressed the invasion and metastasis of MDA-MB-231 and HCC-70 human TNBC cells by promoting the production of KiSS1, a metastasis suppressor [41]. Ferreira et al. found that melatonin exerts its antitumor effects by reducing TNBC cell proliferation, migration, and c-Myc expression [42]. Woo et al. revealed that melatonin enhances tunicamycin‐induced apoptosis in human breast carcinoma MDA‐MB‐231 cells [43]. This contradictory effect may be attributed to the heterogeneity of TNBC. Studies have explored the expression of melatonin receptors (MRs) in TNBC in African-American and Caucasian women, finding that MRs are associated with survival and differentially expressed in these two populations [44]. In our study, we used three TNBC cell lines, BT549, 4T1, and 891, to confirm the role of melatonin and the underlying mechanisms. Our study confirmed the anticancer effect of melatonin on TNBC cells and found that melatonin inhibited TNBC progression through the lnc049808/miR-101/FUNDC1 axis, a ceRNA regulatory mechanism, which has never been reported.

FUNDC1, acting as an activator of hypoxia-induced mitophagy, is an important mechanism for mitochondrial quality control. It sustains oxidative bioenergetics, buffers ROS production, and supports cell proliferation. Li J et al. found the FUNDC1-LonP1 axis could control mitochondrial reprogramming and tumor cell plasticity through transforming between proliferative and invasive states. They demonstrated the role of FUNDC1 in prostate adenocarcinoma, glioblastoma, lung adenocarcinoma, and breast adenocarcinoma [45]. In addition, mitochondria are the core of innate immunity, and aberrant mitochondrial activity leads to immune activation and chronic inflammatory diseases, like cancers. FUNDC1-mediated mitophagy plays a crucial part in the inflammatory response and tumorigenesis [46]. The function of FUNDC1 that we discovered in this study is consistent with the report of Wu L et al., but we disclose for the first time its new mechanism as a ceRNA [47].

In conclusion, our findings indicated that melatonin inhibits TNBC progression via the lnc049808/miR-101/FUNDC1 axis and that melatonin may serve as an alternative therapy for TNBC.

## Materials and methods

### Ethical approval declarations and consent to participate

Approval for this study was obtained from the Ethics Committee of Sun Yat-Sen University Cancer Center Health Authority and was carried out according to the ethical standards of the Declaration of Helsinki. The IACUC of Sun Yat-Sen University Cancer Center approved all animal studies, which were performed according to its guidelines. The availability of data and information on the data set used and analyzed in the current study can be provided by the corresponding author upon request.

### Cell culture and transfection

All cell lines used in this study were obtained from the American Type Culture Collection (USA). All cells were cultured according to the supplier’s instructions and were confirmed to be free of mycoplasma contamination, as verified by DNA fingerprinting. Cells were transfected with Lipofectamine 2000 (Invitrogen, USA). shRNA sequences were synthesized by GeneCopoeia (Rockville, MD, USA) to target Lnc049808 and FUNDC1. A Lenti-Pac HIV Expression Packaging Kit (GeneCopoeia, Rockville, USA) was used to produce lentivirus expressing shRNAs. Puromycin (2 mg/mL) was adopted to select FUNDC1 and Lnc049808 knockdown cells. miR-101 mimics and inhibitors were purchased from GeneCopoeia.

### Microarray and cluster analysis

The TNBC cell lines 4T1 and 891 were pretreated with melatonin (100 nM; Sigma-Aldrich, USA) for 24 h. Total RNA was extracted with TRIzol reagent (Life Technologies, USA). An Affymetrix GeneChip Mouse Genome 430 2.0 Array and Agilent Mouse lncRNA Chip (CapitalBio Technology Corporation, China) were used for microarray analyses. GeneSpring software V. 13.0 (Agilent) was used for quantile normalization and data processing. Heatmaps were generated with Cluster 3.0 software.

### Quantitative RT-PCR analysis

Total RNA was isolated with TRIzol (Invitrogen). The cytoplasmic and nuclear fractions were separated using a PARIS^TM^ kit (Invitrogen). qRT-PCR was carried out in a BioRad CFX96 PCR System (USA) with SYBR Premix Ex Taq II and PrimeScript RT Master Mix (Takara, Japan). The primers were synthesized by Invitrogen (Table S1). An All-in-One miRNA qRT-PCR Detection Kit (GeneCopoeia) was used for qRT-PCR of miRNA. The 2^−ΔΔCt^ method was used to normalize the threshold cycle (Ct) values to those of β-actin or U6.

### CCK-8 and colony formation assays

Transfected cells (1 × 10^3^) were seeded into 96-well plates for 2 days, and then 10 ml/µl CCK-8 solutions (Dojindo Laboratories, Japan) were added to each well. The absorbance at 450 nM was measured after 2 h of incubation at 37 °C. After 2 weeks of incubation at 37 °C, cells (1 × 10^3^) were seeded in six-well plates for colony formation assays. The cell colonies were fixed with methanol and stained with 0.1% crystal violet, and colonies in each well were imaged and counted immediately.

### Transwell assay

A total of 1 × 10^4^ cells were seeded in each well of migration chambers (BD Biosciences, USA), and a chemotactic agent (10% FBS medium) was added to the lower section of the chambers. After 24 h, the cell colonies were fixed with methanol and stained with 0.1% crystal violet, and colonies were counted immediately.

### Mouse xenograft model

A total of 2 × 10^6^ 4T1 cells were subcutaneously injected into the dorsal flanks of 4-week-old female BALB/c nude mice (3 mice per group). After 4 weeks, xenografts were harvested under anesthesia, and tumors were weighed and recorded. For the lung metastasis experiment, 1 × 10^5^ 4T1 cells were injected via the tail vein (3 mice per group). After eight weeks, the mice were euthanized, and the lungs were harvested. Macroscopically visible metastatic nodules were counted and subsequently confirmed by hematoxylin and eosin (H&E) staining.

### Luciferase reporter assay

The FUNDC1 3′-UTR or Lnc049808 sequence (including the miR-101 binding site) was inserted into the pGL3 luciferase vector (Promega, USA) to construct the luciferase reporter vector. A rapid site-directed mutagenesis kit (TIANGEN, China) was used to generate mutations in the seed region of miR-101 as a mutation control. A dual-luciferase reporter gene assay system (Promega) was used to measure luciferase activity.

### RIP assay

Cells were cotransfected with MS2bs-Lnc049808, MS2bs-049808-mt, or MS2bs-Rluc, and MS2bp-GFP. After 48 h, a Magna RIP RNA-Binding Protein Immunoprecipitation Kit (Millipore) was used to conduct RIP. The level of miR-101 was measured after the RNA complexes were purified. The Ago2 RIP assay was performed using an anti-Ago2 antibody (Millipore), and the levels of lnc049808, FUNDC1, and miR-101 were further measured.

### Statistical analysis

All data were analyzed with SPSS 19.0 software. We used *t*-tests and Pearson *χ*^2^ tests to compare the differences between groups. Unless otherwise stated, the data are expressed as the mean ± standard deviation of three independent experiments. *p* < 0.05 was considered statistically significant.

## Supplementary information

Table S1

## Data Availability

The availability of data and information on the data set used and analyzed in the current study would be provided by the corresponding author upon request.
